# 

**Published:** 1850-09

**Authors:** 


### CONTENTS

OF NO. III.

---

### ORIGINAL COMMUNICATIONS.

### ESSAYS AND CASES.

Art. I.—A Report on Typhoid Fever made to the Scott County Medical Society. By W. L. Sutton, M. D., of Georgetown, Ky. - - - - - - - 185

### REVIEWS.

Art. II.—A Systematic Treatise, Historical, Etiological, and Practical, on the Principal Diseases of the Interior Valley of North America, as they appear in the Caucasian, African, Indian, and Esquimaux varieties of its Population. By Daniel Drake, M. D. - - - 228

### SELECTIONS FROM AMERICAN AND FOREIGN JOURNALS.

Treatment of Internal Hemorrhoids, - - - - - 257
On the alleged frequency of Ulceration of the Os and Cervix Uteri—Speculum Practice, - - - - 258
Febrifuge and Antiperiodic Properties of Chloroform, - - 265
Why Epidemics Rage at Night, - - - - - - 265
Mortality of Cities, - - - - - - - - 266
On Incontinence of Urine in Children, - - - - - 266
Probable Danger from the use of Cod-Liver Oil, - - - 268

ORIGINAL INTELLIGENCE.

Death of Dr. Troost, - - - - - - - 269
The President of the Philadelphia County Medical Society, - 271
The New Hampshire Journal of Medicine, - - - 272
Jarvis's Adjuster, - - - - - - - - 272
Dr. Sutton on Typhoid Fever, - - - - - - 273
Transactions of the Medical Association of Southern Central New York, at the annual meeting held at Elmira, June, 1850, - - - - - - - 273
Report of the Trial of the People vs. Dr. Horatio N. Loomis, for Libel, - - - - - - 274
New Remedies, - - - - - - - - 274
Changes in Medical Schools, - - - - - - 275
Fungus Hematodes, - - - - - - - - 275
Books Received, - - - - - - - 276

